# Fast mapping in hominids

**DOI:** 10.1007/s10071-025-01974-x

**Published:** 2025-07-01

**Authors:** Dahliane Labertoniere, Vanessa A. D. Wilson, Carla Pascual-Guàrdia, Katrin Skoruppa, Klaus Zuberbühler

**Affiliations:** 1 Department of Comparative Cognition, Institute of Biology, University of Neuchâtel, Neuchâtel, Switzerland; 2 Institute of Speech and Language Therapy, University of Neuchâtel, Neuchâtel, Switzerland; 3 Department of Comparative Language Science, University of Zürich, Zürich, Switzerland; 4 Center for the Interdisciplinary Study of Language Evolution (ISLE), University of Zürich, Zürich, Switzerland; 5 School of Psychology and Social Work, University of Hull, Hull, UK; 6 School of Psychology & Neuroscience, University of St Andrews, St Andrews, UK; 7 Laboratoire de Sciences Cognitives et Psycholinguistique, ENS, PSL University, Paris, France

**Keywords:** Fast mapping, Word learning, Language evolution, Comparative cognition, Language acquisition, Meaning, Mental representation

## Abstract

Fast mapping is essential when children acquire language, but whether the required cognition is uniquely human or shared with animals is debated. Although documented in dogs and cats, both species have a history of domestication of social cognition, so that it remains unclear whether fast mapping is naturally present in non-domesticated animals. Here, we used an eye-tracking paradigm to test three species of hominids – gorillas, orangutans and humans – in their ability to rapidly learn to associate novel sounds with objects in their everyday noisy environment. The task was difficult for all participants, but while adult humans showed evidence of fast mapping, we could not detect any sign of learning in the other hominids. These species differences could have trivial causes, such as problems with attention or motivation, but it is also possible that fast mapping requires a preexisting lexicon before becoming an effective learning mechanism, or that it has simply evolved after the shared ancestor of all great apes.

**Supplementary Information:**

The online version contains supplementary material available at 10.1007/s10071-025-01974-x.

## Introduction

Fast mapping is a learning ability regarded as essential during language acquisition in human children. It is defined as the ability to form hypotheses about the meaning of a new form (a spoken or gestural unit), often after only one or a few exposures (Carey 1978) and is often seen as the first step in the word learning process. Fast mapping is based on the association of a form – typically a sound – to a referent, typically an object in the child’s surroundings. Fast mapping is probably essential in the acquisition of lexical meaning and could explain how children efficiently learn their immense vocabulary of tens of thousands of words in just a few years (Bloom 2002).

In empirical studies of fast mapping, children will typically be exposed to a few instances of a new word in conjunction with a referent and subsequently tested to see if they correctly learnt the association (Heibeck and Markman 1987; Behrend et al. 2001; Vlach and Sandhofer 2011). In some cases, children will be retested a few weeks later to check for signs of retention (Carey 1978; Vlach and Sandhofer 2012), a memory consolidation mechanism that is, along with generalisation, involved in building the lexicon (Vlach and Sandhofer 2012) after the first step of fast mapping. Although fast mapping is important during language acquisition in children, human adults continue to deploy this ability, as demonstrated by various word learning studies (Bloom 2002; Halberda 2006; Markson and Bloom 1997; Vlach and Sandhofer 2012).

There is an ongoing debate whether fast mapping is specific to language acquisition or whether the mechanism is cognitively more general and also relevant in other domains (Markson and Bloom 1997; Behrend et al. 2001). In the latter case, this might suggest that fast mapping evolved before modern humans and should therefore be present in some other animal species, which face similar challenges when establishing various sound-event relations during ontogeny (León et al. 2022).

Relevant here is evidence for fast mapping in dogs (*Canis familiaris*). In one study (Kaminski et al. 2004), a border collie was able to form novel word-object associations, based on an exclusion learning paradigm. In the experiment, the dog encountered an unfamiliar object within an array of familiar items after a ‘fetch’ command by his owner uttered in conjunction with an unfamiliar word. The dog then preferentially retrieved the novel object and, moreover, showed evidence for retention when retested a few weeks later. In a more recent study (Fugazza et al. 2021), two dogs (a border collie and a Yorkshire terrier) were tested on their ability to choose between two newly learnt object labels. First the dogs were exposed by their owners to two new toys associated with novel labels, then the owners asked the dogs to bring back one of the novel toys, labeling it. Both individuals performed above chance in this task, specifying the conditions under which fast mapping operates in dogs. Specifically, the dogs learnt the new labels while playing with their owners, who explicitly labeled the objects, and both dogs were already very familiar with object-name learning. Importantly, 20 other family dogs that had no object-naming experience failed the task.

There is also evidence for fast mapping in domestic cats (*Felis catus*). Here, in a habituation-dishabituation manual gaze-tracking experiment (Takagi et al. 2024), cats were first exposed to a novel word-object association during the habituation phase and then responded more strongly to the incorrectly labeled object than to the correctly labeled object in the dishabituation phase (e.g. they looked longer at a red object paired with the label which had been previously associated with a blue object), indicating that they had associated the stimuli with their correct labels.

These studies suggest that fast mapping is not limited to humans and might also exist in other species. However, the caveat with this interpretation is that cats and dogs are domesticated species that have evolved to interact with humans in their environments (Hare et al. 2002). Whether fast mapping is naturally present in non-domesticated animals is still unknown. We are unaware of any research that has examined this question amongst species that share phylogenetically close common ancestors with humans – the typical starting point to examining the origins of language (Hauser et al. 2002).

Referentiality, a key feature of human language which is central to fast mapping, has been observed in other species under limited contexts. In addition to observations that language-trained apes can use referential labels (see the studies on Kanzi, e.g. Savage-Rumbaugh et al. 1986), a small but mounting pool of evidence suggests some, albeit limited, use of referential calls and gestures in untrained apes (Watson et al. 2015; Slocombe and Zuberbühler 2005; Genty and Zuberbühler 2014). Whilst claims of naturally occurring referentiality in apes are still debated (León et al. 2022; Pepperberg 2021; Fischer et al. 2015), current evidence indicates that differences in referentiality between humans and nonhuman apes is one of degree.

To address this, we used an eye-tracking paradigm (a method that was successfully used in both humans and nonhuman apes, see Wilson et al. 2024; Kano et al. 2012; Kano and Call 2014) that allowed us to directly compare human adults and nonhuman great apes in a simple fast mapping task. We used a protocol similar to one used in infant studies (see Labertoniere et al. 2025; Schafer and Plunkett 1998), requiring no explicit learning. To improve the ecological validity of the study, we did not follow the standard protocol, which is to present objects in front of simple, featureless backgrounds to enhance their salience (e.g. Schafer and Plunkett 1998). Instead, objects were presented how they are usually encountered by human and ape infants in real life learning situations, that is, embedded in a complex, visually-structured natural background that sometimes changes between each encounter. This presentation is important because of evidence suggesting that the nature and variability of the background can impact learning (Axelsson and Horst 2014; Twomey et al. 2018; Vlach and Sandhofer 2011; Labertoniere et al. 2025), and that input variability facilitates the generalisation of novel concepts (Bourgoyne and Alt 2017). In human infants in particular, background variability and change in target object orientation seem to promote learning (Labertoniere et al. 2025).

Here, we explored fast mapping in three species of hominids, human adults, gorillas (*Gorilla gorilla*) and orangutans (*Pongo abelii*) and how learning was influenced by the variability in the background on which the referents were presented. If fast mapping evolved before language, we expected to find evidence in all three species. If variation in background mattered, we expected it to make learning less difficult (Bourgoyne and Alt 2017; Labertoniere et al. 2025).

## Method

### Participants

#### Great apes

N = 4 gorillas (3 F, mean age = 8.2 years, range = 2.8$-$15.9 years) and N = 1 orangutan (M, age = 19.9 years) served as participants and were tested in their indoor enclosures at Basel Zoo, Switzerland. All contributed to the experiment voluntarily, could leave at any time and were never separated from the rest of the group or food deprived. Participation was rewarded with sugar-free syrup, in amounts approved by the zoo’s veterinary team. Since participants were not separated from their peers, social distractions were common, simulating the conditions under which learning takes place in nature.

#### Humans

N = 5 French-speaking bachelor students (mean age = 22.4 years, range = 19–26 years, gender/sex was not recorded) at the University of Neuchâtel, Switzerland, served as participants following informed consent. To provide a learning environment similar to the one experienced by the apes at the zoo, we collected the data during an ongoing lab visit during which some of the participants’ peers walked by and talked, which likely generated comparable social distraction.

#### Stimuli

To investigate whether participants could rapidly learn novel sound-object associations, we presented them with four different yoked pairs of objects associated with four different yoked pairs of sounds. Objects were pictures of exotic fruits, presented on four different naturalistic backgrounds (rocks, leaves, soil, grass) as it might happen in real-life. We chose exotic fruits as objects to keep apes interested and to minimise the risk that humans already had a name for them. See all pairs of objects in Supplementary Table 2, and see Supplementary Figure 1 for one pair of objects on all possible backgrounds. As sound stimuli, we generated pseudo-words that complied with French and Swiss-German phonotactic rules (e.g. /if/, /ovo/), produced by a bilingual speaker of both languages. While designing the experiment, we considered using modified ape calls as pseudo-words. However, since we were testing multiple species and both nonhuman test populations were habituated to human language (specifically, Swiss-German) through zoo keepers, we decided to stick to human pseudo-words. Humans were to be tested in a French-speaking part of Switzerland. See Supplementary Table 1 for a list of all paired pseudo-words.

### Procedure and apparatus

For the apes, gaze was recorded at a distance of approximately 60 cm with a Tobii Spectrum eye-tracker (300 Hz sampling rate), positioned under a monitor (23.8”, 1920 x 1080 pixels), which displayed the images using Tobii Pro Lab. The pseudo-words were played by a loudspeaker (Fostex 6301B 10W amplifier) hidden behind the screen. The experiment was coded in Tobii Pro Lab.

For N = 2 gorillas, the apparatus was positioned in a small enclosure separated from the main enclosure by a sliding door (open during testing) and a plexiglass window, with the experimenter present with access to controls, see Supplementary Figure 2. For N = 2 gorillas and the orangutan, the apparatus was part of a mobile setup, consisting of a height-adjustable, purpose-built plexiglass box, which could be placed in front of the enclosure, see Supplementary Figure 3. In this setup, a drinking nozzle attached to the vertical mesh ensured that eyes remained positioned between the mesh to avoid interference with gaze detection.

For the humans, gaze was recorded in the same way with a Tobii Pro X3-120 eye-tracker (120 Hz sampling rate) positioned under a monitor (Dell, 24.0”, 1920 x 1080 pixels). Here, the pseudo-words were presented via two stereo loudspeakers (Fostex PM0.5n, 70W amplifier) hidden under the table supporting the monitor. This setup was placed inside a sound-attenuated booth with the door open to allow for distractions. The script running the experiment was coded in Matlab version 2019b (The Math Works, Inc., 2019) using the Psychtoolbox (Brainard and Vision 1997; Pelli 1997; Kleiner et al. 2007) and the Titta toolbox (Niehorster et al. 2020). The experimenter had access to the controls from outside the booth. To match the ape condition as closely as possible, human participants were told that they could pause or abort the experiment at any time (none did) but otherwise they were not given any instructions. Also important, in contrast to similar experiments (e.g. Markson and Bloom 1997), we decided not to use any carrier sentences or attention getters (e.g. "hey" or "This is a xxx"), as they would be meaningless to nonhuman apes and would only give humans an unfair advantage.

For the human participants, we carried out a full 5-point calibration with pulsating dots before starting the experiment. For the ape participants, a session began with a green screen displayed for 2 s and a 4-point validation of a previously recorded calibration. This calibration was recorded prior to the testing sessions following an established protocol in Wilson et al. (2024): we installed a drinking nozzle so that the participant’s head ended up in a central position in front of the screen, and once a participant was properly positioned, we carried out a 2-point calibration.

### Experimental design

The entire experiment consisted of four sessions (two sessions with invariable backgrounds and two sessions with variable backgrounds). Each session, which corresponded to one specific pair of object–pseudo-word, consisted of six repeated blocks. One block lasted at least 23 s, as follows.

#### Invariable background (Pairs 2 and 3)

Each block started with a first 6000 ms *learning phase* during which one exotic fruit was presented with the same background three times in a row, each time for 2000 ms accompanied by the same pseudo-word, 500 ms after image onset. This first learning phase was then followed by a *second learning phase* during which another exotic fruit was presented in the same way, again accompanied by its own pseudo-word. The two paired fruits were chosen to be maximally different in size, colour and shape. We also made sure that the two paired pseudo-words differed in the number of syllables and were as phonetically distant as possible from each other.

The first and second learning phases were separated by a gaze-contingent (manual for the nonhuman apes, automatic for the humans) visual attention getter (AG).

Provided the participant paid attention to the screen during a second attention getter, this was then followed by a *first test phase*, during which the two fruits appeared side-by-side for 2500 ms without any sound. Subsequently, an attention getter was presented between the two fruits for 500 ms to re-center the participant’s gaze. Immediately after this AG, the two fruits were shown for another 2500 ms, but this time with one of the two pseudo-words broadcast 500 ms after stimulus onset. This phase was then followed by the *second test phase*, identical to the first one, during which the *other* pseudo-word was broadcast.

Once a block (i.e., two learning and two test phases) was completed, it could be repeated five times, up to block six (depending on the participant’s motivation). The order of presentation of the stimuli was changed between blocks. A participant who had completed all six blocks in a session had received N = 18 exposures to the two novel fruit-word associations during the learning phases and had been tested N = 6 times on each association during the test phases.

#### Variable background (Pairs 1 and 4)

**Fig. 1:**
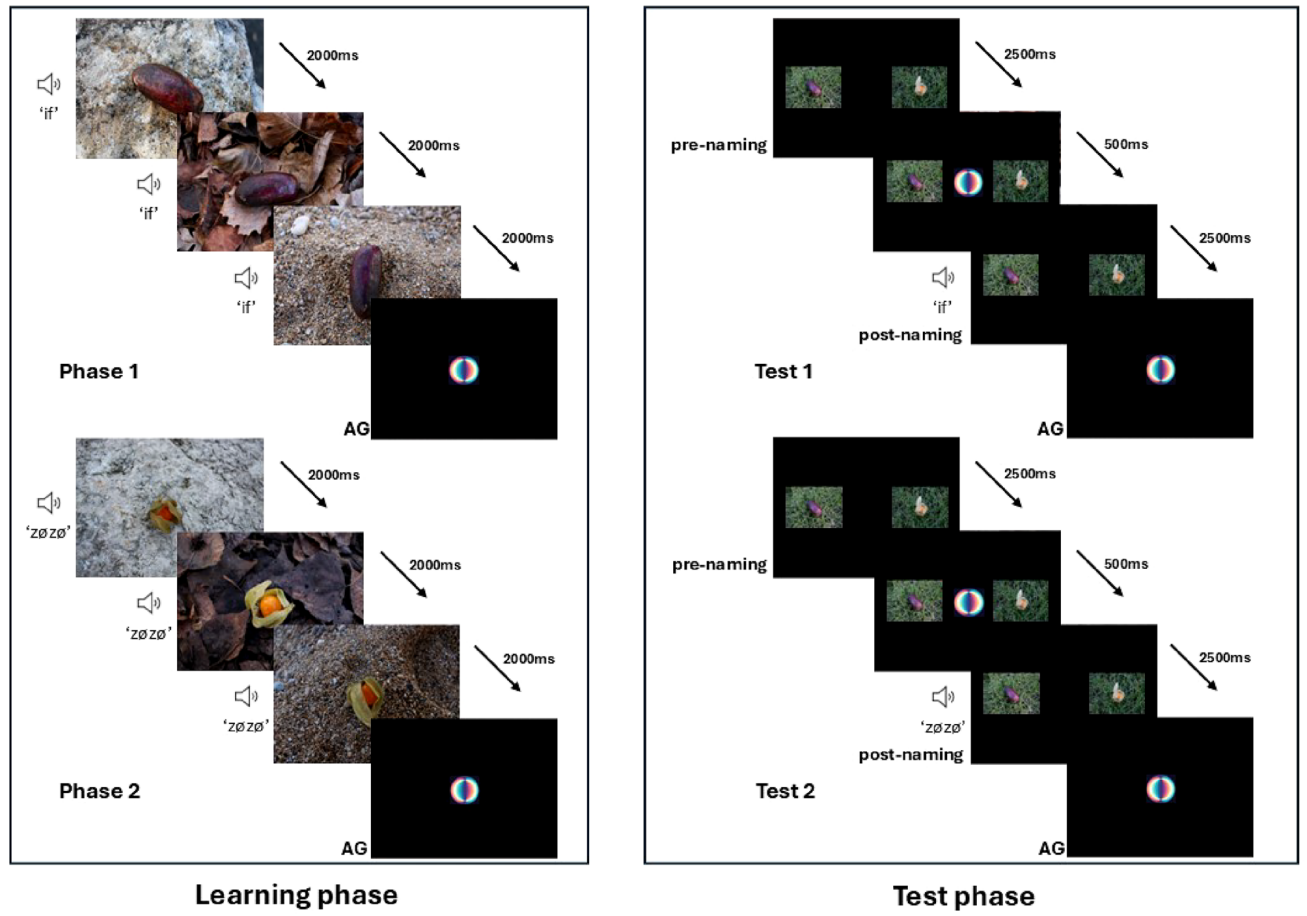
Schematic representation of the experimental design for one block in the variable background condition. The same block is repeated five times with a change in the order of the stimuli to form one session (here corresponding to Pair 1). For the invariable condition, each fruit would be presented on the same background and at the same angle during Learning and Test

To test the impact of the visual environment on learning, the same design was used, but this time the fruit was presented on four visually different backgrounds and photographed from different angles for each of its appearances during the learning and test phases (see Supplementary Figure 1). Figure 1 recapitulates the procedure schematically for the variable background condition.

## Data analysis

For ape participants, data files supplied by Tobii Pro Lab were transformed in Matlab for subsequent analyses in R Studio (running R version 4.2.2). Our files provided gaze hits in our two areas of interest (AOIs), the left and right images in the test, every 3 ms. AOIs were defined as the area around an image – a 350*620 px rectangle. For human participants, data analysis was carried out similarly, except that raw data were supplied directly in Matlab format. We used a Matlab script to transform those raw files into files providing gaze hits in the two AOIs every 8 ms. For both species, we computed the proportion of target looks for the pre- and post-naming phases of each trial.

Human participants completed all four sessions of the experiment on the same day, with short pauses between sessions (for a total duration of approximately 15 min). The apes often struggled to remain focused, but still completed up to two sessions per day, depending on motivation. One subject had to come back two days later to finish one of her block. We excluded test trials where the participant did not pay any attention to the screen (2) or where the sound was not playing due to technical issues (3). In both cases, the experiment was stopped and the block run again at a later time.

If fast mapping took place, we predicted relatively increased looking times towards the fruit whose pseudo-word was played during the (first or second) test phase. Specifically, we predicted looking time to the named fruit to increase between the pre- and the post-naming phases in the test phases, expressed in terms of mean proportion of target looks (PTL).

## Results

### Great apes

We managed to record gaze data in N = 130 of 216 test trials (60.2%) in the pre- and/or post-naming phases. For the exact number of trials per session provided by each participant, see Supplementary Table 3. Some participants did not go up to five repetitions of a block. Since the data were not normally distributed (Shapiro-Wilk test: W = 0.798, *p* < 0.001) but had a zero–one inflated beta distribution, we applied a slight shrinkage to the proportional data (zeros were transformed to 0.0001 and ones to 0.9999). We built a generalised linear mixed model for beta distributions with a logit link using the glmmTMB package (Brooks et al. 2017). PTL was the dependent variable, with ‘background’ as a fixed effect, ‘naming’ (pre- vs. post-) as a nested fixed effect within ‘background’ and ‘participant’ as a random effect. The factor ‘background’ was sum-contrasted for the model. Adding block number did not improve the model, so it was not included in the final model, which was as follows:

- PTL $\sim$ Background / Naming + (1 | Participant)

**Fig. 2:**
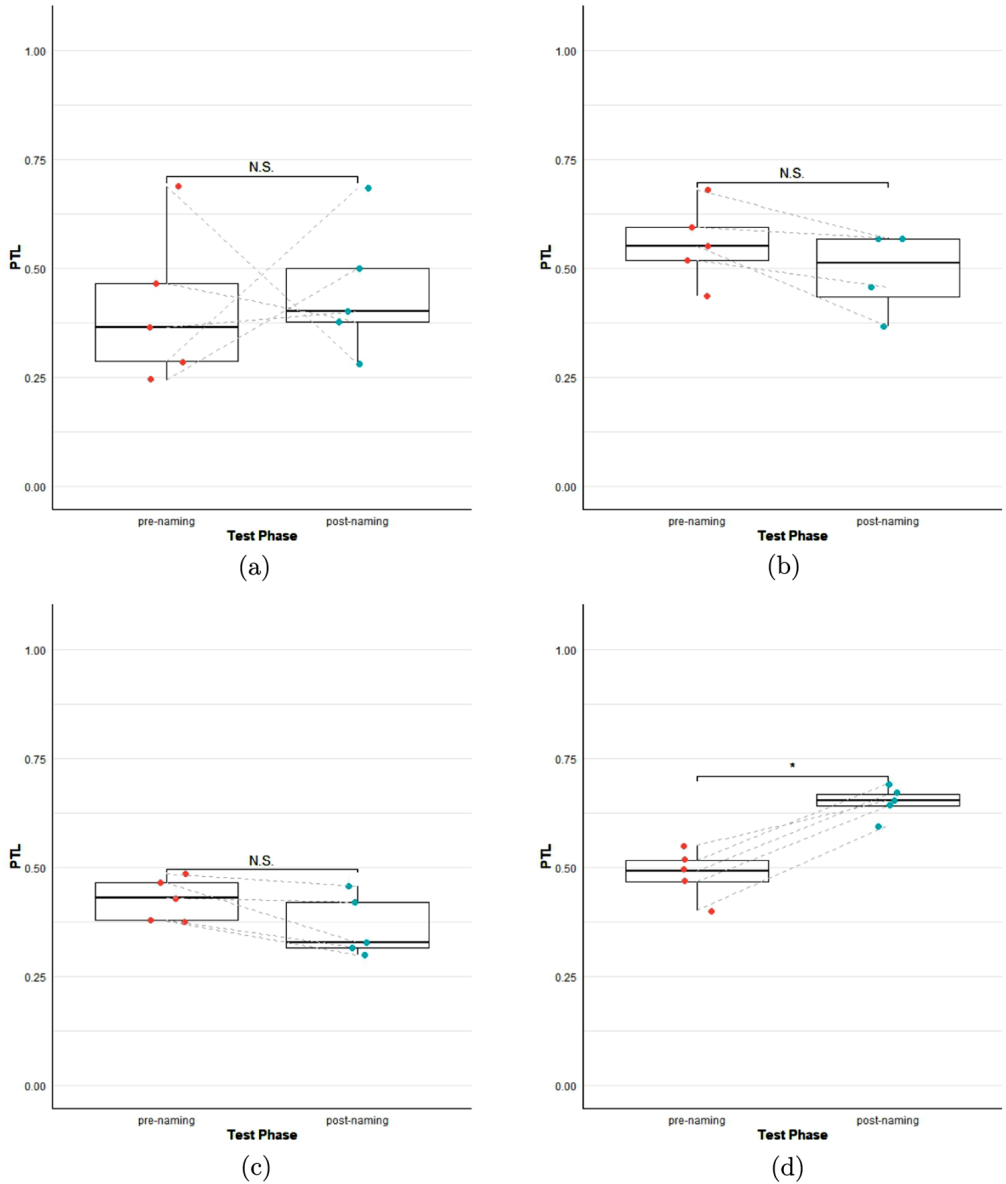
Mean gaze proportions in pre- and post-naming phases in the variable condition (**a**,** c**) and invariant condition (**b**,** d**) for apes (**a**,** b**) and humans (**c**,** d**). Boxes represent the interquartile ranges (IQR), horizontal lines depict medians, whiskers represent minimum and maximum values that do not exceed 1.5 x IQR, and dots represent individual subjects. Asterisks indicate a significant difference (*p* < 0.05)

We found no significant increase in looking time between pre- and post-naming phases, neither with invariable nor with variable backgrounds (see Table 1). A graphical overview of the data (with PTL averaged by subject) showing comparisons between pre- and post-naming phases of both background conditions can be found in Fig. 2 (a, b).

**Table 1:** Results of the models looking into the presence of a naming-effect in both conditions in human and nonhuman great-apes

Predictor	Estimate	SE	*p*
Nonhuman apes			
(Intercept)	0.0101	0.131	0.939
Background	$-$0.150	0.132	0.255
Background_Variable:Naming	0.038	0.305	0.901
Background_Invariable:Naming	$-$0.321	0.330	0.330
Humans			
(Intercept)	$-$0.089	0.092	0.330
Background	$-$0.075	0.092	0.415
Background_Variable:Naming	$-$0.292	0.179	0.103
Background_Invariable:Naming	0.577	0.180	**0**.**001**

*SE* standard error

### Humans

Participants provided a total of N = 240 test trials (N = 24 per background condition per participant). As with the apes, data were not normally distributed (Shapiro-Wilk test: W = 0.877, *p* < 0.001) but followed a zero–one inflated beta distribution. We carried out operations analogous to the ape data and set up a model using PTL as the dependent variable, ‘background’ as a fixed effect, ‘naming’ (pre- vs. post-.) as a nested fixed effect within ‘background’, and ‘participant’ as random effect. Adding block number did not improve the model and was not considered any further.

We found a significant main effect of naming ($\beta$ = 0.577, SE = 0.180, *p* = 0.001), although in the invariable background condition only (see Table 1). Figure 2 (c, d) shows comparisons between pre- and post-naming PTL averaged by subject for both background conditions.

## Discussion

Our study aimed to explore fast mapping in humans, gorillas and orangutans. When tested for their ability to associate a novel sound with a novel object, only humans showed an increase in looking time towards the correct object after its label was played back. Furthermore, they managed to learn novel associations only when the visual environment in which the objects were presented was stable. No evidence of a learning effect was observed in the non-human apes.

How a signal obtains its meaning is of cross-disciplinary interest, particularly in the areas of language acquisition and evolutionary studies of cognition. A key mechanism is fast mapping (Carey and Bartlett 1978), a mental process by which a representation is formed following exposure to a referent in conjunction with a signal. Fast mapping likely plays an important role in word learning and provides an explanation for the astonishing speed at which children acquire their vocabularies. Although animal communication also involves learning (Vernes et al. 2021), the problem is somewhat simplified by the fact that most communication systems consist of limited signal repertoires, usually produced as part of biologically fixed functions (Oller et al. 2013). Nevertheless, when hearing an unfamiliar signal produced by a more experienced conspecific, the problem for an infant ape is identical to that of a human child, that is, to identify which aspect of reality the signal refers to and to retain this relation for the future.

The current literature is equivocal as to the nature of fast mapping. Either it is simply a special case of cognition based on one-trial learning, for which there are countless examples in the animal literature, ranging from conditioned taste aversion (Garcia et al. 1955) to rapid associations of alarm calls to arbitrary referents (Curio et al. 1978; León et al. 2022). Alternatively, fast mapping may be a mechanism specifically evolved for word learning (Behrend et al. 2001) or a mere byproduct of it. Although analogues of fast mapping have been demonstrated in dogs and cats, both species are domesticated, and dogs in particular have undergone strong selection facilitating interspecies communication with humans – the domestication of social cognition (Hare et al. 2002). At the same time, we are not aware of systematic research on spontaneous fast mapping in non-domesticated animal communication, including humans’ closest relatives, the great apes.

We addressed this limitation by directly comparing human and nonhuman great apes in a fast mapping paradigm based on eye-tracking. We chose this method to facilitate direct cross-species comparison using one type of data. We also made an effort to test participants under equivalent conditions and with ecologically relevant stimuli, i.e., an unfamiliar food item encountered on its natural substrate of varying visual complexity. To this end, participants experienced three exposure trials during which an unfamiliar fruit was paired with its pseudo-word, a simple (di- or tri-)syllabic sound sequence. We subsequently tested each participant on whether they had been able to retain the association, by presenting pairs of fruits whilst playing the name of one, predicting that participants’ gazes should be guided by the previously established sound-referent relation.

We found that the task was difficult for the participants of all three species. Adult humans, however, showed some evidence of fast mapping of novel sounds to novel objects. Interestingly, however, this learning only happened if the fruit was presented on an invariable background, with no evidence for learning if the background changed across the N = 6 learning trials. This finding went against our initial prediction, i.e., that encountering new objects in variable ways enhanced generalisation and concept formation. Nevertheless, the fact that we found a learning effect, even though we deliberately made the task difficult with a distracting social environment and lack of explicit instructions, shows that, in principle, fast mapping is possible with our paradigm. In contrast, we found no evidence for fast mapping the association of an acoustically distinct utterance and a specific fruit in great apes. Bearing in mind that it is difficult to draw firm conclusions from negative evidence, we can think of the following explanations for this apparent human-ape divide.

### Limitations

To start with, several limitations of our study could explain our results. First although the task appeared to be cumbersome for all species, it appeared to be even more difficult for the apes to remain focused, which may have limited their ability to learn novel associations. The sample sizes were exceedingly small for both human and nonhuman participants (the human sample size was chosen to match the ape one), suggesting that a larger participant pool might have washed out eventual outliers due to low motivation. It is also possible that apes would have needed more training instances to elicit learning (like some children sometime receive, see Kan and Windsor 2010), although such additional training would have likely only worsened their already prevalent motivation problem (Wilson et al. 2023). The number of learning exposures did not play a role in the adults’ ability to fast map, suggesting that the apes’ underperformance had other roots.

Second, and related to the above, we noted that the stimulus set was generally not particularly effective in eliciting learning. Although human participants could learn new associations if the background did not change, such learning did not occur if the background changed. Interestingly, this effect appears to be different in 14-20-month-old children tested in a similar paradigm (Labertoniere et al. 2025). Here, learning was only observed when the background changed, which is arguably much closer to what children experience in real-life word learning situations. The adult participants probably guessed that the goal of the experiment was to test for learning of novel names for objects, which might have incentivised them to look towards the object indicated by the pseudo-word during the tests. Here, the invariant background condition might have been more effective in revealing the goal of the experiment, suggesting that participants performed better because less attention was needed. Interestingly, for adults learning new languages, explicit teaching strategies appear to be more effective (DeKeyser 2003), suggesting that word learning strategies change during ontogeny from childhood to adulthood.

Third, the conditions in which our experiment took place were rather artificial. Even though we used pictures of real objects with naturalistic backgrounds in order to improve ecological validity, they were still presented on a screen in an artificial situation. One way to improve this paradigm further would be to conceive a task using real objects (more similar to the task in Kaminski et al. 2004). It is also possible that adult humans were more familiar with the testing situation (looking at pictures on a screen). However, while adult humans are very used to this kind of experimental setup, most of the apes tested in Basel zoo are also well accustomed to monitors, having participated in many touchscreen and eye-tracking studies since 2018 (e.g. Brocard et al. 2024; Wilson et al. 2024). Because of this experience, even if there was a difference in familiarity between all tested species, it should be minimal.

Fourth, it is possible that fast mapping did take place in the apes but that our way of assessing learning was simply not fine-grained enough. It is important to note that, even when the apes were focused on the task, their total looking time to the screen was short (for the apes: a mean of 965 ms in pre-naming and 548 ms in post-naming; for the humans: a mean of 1814 ms in pre-naming and 2178 ms in post-naming). This short duration could have masked a potential learning effect in the pre- vs post- attention calculations (and might also indicate that humans were in general more engaged and interested in the stimuli). In a typical trial, apes quickly scanned the stimuli but then preferred to look elsewhere, a pattern also found in other great ape studies (Kano and Tomonaga 2011; Wilson et al. 2024). One way to address this issue might be to assess participants with forced choices on a touch screen, to make the task more interactive and rewarding (see Brocard et al. 2024; Wilson et al. 2023).

### Other possible explanations

In addition to these limitations, a few other factors could play a role. First, while the learning situation modelled in our experiment is likely common in real life, and certainly ecologically relevant (a referent is spotted by an individual, who utters speech and then shares attention with a bystander, allowing the bystander to form an association between sound and referent, and to remember it), it is not clear whether such situations also occur in wild animals. One documented domain in which this behaviour has been observed is in the context of anti-predator behaviour, when young individuals rapidly learn to apply one of their species-specific alarm calls to a new referent (Deshpande et al. 2022; León et al. 2022), suggesting that events involving some sort of danger might have been more effective in triggering fast mapping. Regarding our design, we are not aware of any systematic research investigating how primates or other animals learn to use vocal signals to refer to food.

Second, it is possible that the nature of the stimuli, in our case spoken pseudo-words, promoted learning in humans, but not in great apes. Relevant here is that captive orangutans respond better to their keepers’ gestures compared to words (Dezecache et al. 2019), suggesting that human speech sounds may not be optimal for comparative fast mapping research. In another relevant experiment (Déaux et al. 2021), chimpanzees were faster to learn in a touchscreen-based visual discrimination task if the images were paired with conspecific food calls as opposed to other sounds or with silence, suggesting that some sound-referent associations appear to be more natural than others, with conspecific, referentially correct signals most likely being more efficient than other sounds. Arguably, it may have been fairer towards the gorillas and orangutans to use stimuli crafted from their own species-specific vocalisations, but this manipulation would have introduced other unwanted biases. For instance, gorilla and orangutan vocalisations already possess natural functions (e.g. Luef et al. 2016), so that producing novel versions of such signals would have generated an unnatural and possibly disturbing experience for the subjects. Also, since we were keen to use the same stimuli for all subjects, this addition would have created a complex study design with nine experimental conditions. In the end, we decided to use human vocalisations (pseudo-words) only, based on the fact that all three species were very familiar with human speech. Specifically, the great apes at Basel Zoo were used to hearing Swiss-German from interacting with the the zoo keepers. Presumably, they were thus as familiar with these sounds as our human subjects. Regarding the objects, as interest in the category of the stimuli promotes label learning in children (Ackermann et al. 2024) and could also support associative learning in animals (e.g. the dogs learnt labels for new toys – objects that they probably were quite interested in – in Fugazza et al. 2021; Kaminski et al. 2004), using objects relevant and interesting to the focus species seems key. Food items seemed such a category to us, but our stimuli might have not been salient enough or recognised as food by our subjects. Future studies might seek to present the novel sound-object associations during brief video clips, which tend to increase attention in apes compared to still images (e.g. Wilson et al. 2024), and perhaps to switch to animate objects or to at least present the fruits with some sort of natural motion.

Third, fast mapping may require crucial ontogenetic experience to scaffold on. For instance, the fact that it has been observed in domesticated animals suggests that the ability has emerged from individuals having had prolonged contact with humans, which might have also led to a small lexicon of words (see Takagi et al. 2024). Whether this experience is the main explanation for the species difference is doubtful, mainly because fast mapping also seems to be involved in domains other than language learning (Markson and Bloom 1997; Behrend et al. 2001).

Finally, the language acquisition literature is full of evidence highlighting the important role of social interactions during learning. A particularly effective situation is when two participants share their attention towards a referent whilst the name is produced (Tomasello and Farrar 1986). Specifically, children can learn novel consonant contrasts when tutored by a person, but not from a video recording of the same person (Kuhl et al. 2003). Human adults also seem to benefit from learning in a social context (Jeong et al. 2010) and in all likelihood similar processes play a role in great apes (Howard et al. 2017). In our design, it was not the case that the context was social since the name was broadcast from a speaker, with no real-life reference provider present. This stimulus presentation may in fact have made learning harder for all participants, and explain that fast mapping did not seem to happen in the apes. However, many fast mapping studies with human infants show positive results using this kind of protocol. Crucially, both studies on dogs (Kaminski et al. 2004; Fugazza et al. 2021) in which fast mapping was reported were conducted in socially more adequate situations. Not only were the dogs already familiar with the process of new label learning from early puppyhood, the experiment was embedded in a deeply social setting, with dogs and owners playing and interacting, including joint attentional episodes. In the cat study (Takagi et al. 2024), there was also a social component, as the sound stimuli were spoken by their owners. Indeed, cats failed to fast map in the same experiment when sound stimuli were electronic sounds.

## Conclusion

We demonstrated that our very basic design was sufficient to trigger fast mapping in humans, but that various factors might have made it more difficult for great apes, including species differences in motivation, attention, the effectiveness of the stimuli and the importance of social interaction. Until these hypotheses have been addressed by future research the tentative conclusion is that spontaneous fast mapping of novel names for objects in the environment, and the concept formations that go along with this process, is a uniquely human process not naturally present in non-domesticated animal communication. The fact that some domesticated species have provided positive evidence, however, suggests that fast mapping does not essentially require a primate brain. It also suggests that fast mapping does not evolve along phylogenetic branches, but emerges as a consequence of special social cognition in which learning takes place during directed social interactions rather than the default way of observing signal-referent relations in the surroundings. Future research is likely to produce progress on elucidating these factors, and to provide more of a definite answer about the status of what appears to be a human universal.

## Supplementary information

Supplementary files can be found in a separate pdf file.

## Supplementary Information

Below is the link to the electronic supplementary material.

Supplementary file 1 (pdf 7983 KB)

## Data Availability

Data files and code can be shared with interested parties by contacting the first author.
