# Supplementary material for: Fast mapping in hominids
Source: Anim Cogn. 2025 Jul 1;28(1):53. doi: 10.1007/s10071-025-01974-x (PMC12213842; doi:10.1007/s10071-025-01974-x)
Supplement: Supplementary file 1 — (pdf 7983 KB) [file 10071_2025_1974_MOESM1_ESM.pdf]

Supplementary Information

Supplementary Table 1: Pairs of pseudo-words used in the experiment

Pair 1	/if/	/zøzø/
Pair 2	/fa/	/ømø/
Pair 3	/yʃ/	/vuvu/
Pair 4	/za/	/ovo/

Supplementary Table 2: The four pairs of objects on the four different backgrounds

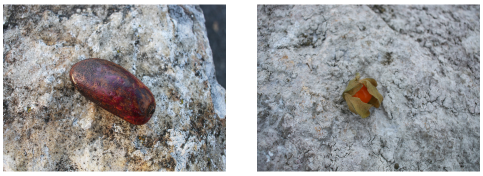

Pair 1

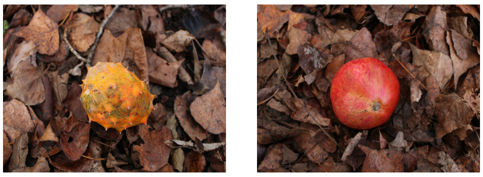

Pair 2

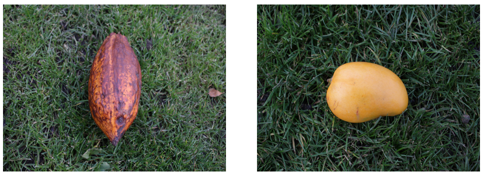

Pair 3

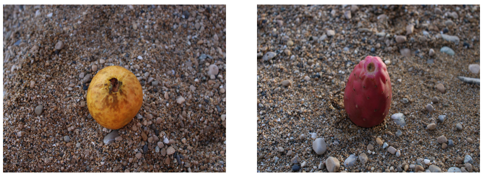

Pair 4

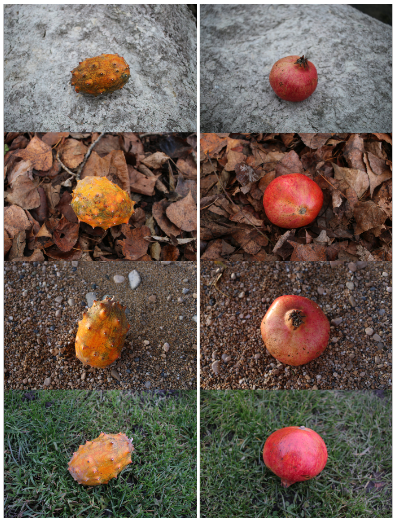

Supplementary Figure 1: One pair of objects with different orientations on the four different backgrounds

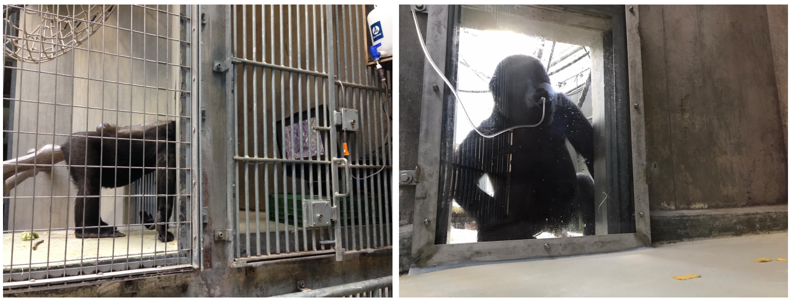

Supplementary Figure 2: Enclosure setup

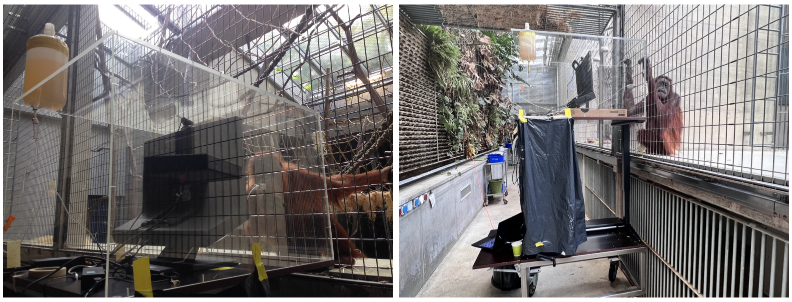

Supplementary Figure 3: Mobile setup

Supplementary Table 3: Number of test trials each ape provided data for per session

	Session 1	Session 2	Session 3	Session 4
Subject 1	8	7	6	6
Subject 2	7	6	2	1
Subject 3	12	10	11	2
Subject 4	5	11	9	10
Subject 5	6	5	6	0

Supplementary data for the nonhuman great apes:

Since we lost a number of trials in which the apes either did not pay attention during the test phase or the eye-tracker could not register their gaze, we decided to collect more data for the N=4 gorillas, from one week to three months after they had completed a session (therefore they repeated some sessions). When running the model on these data combined with the previous test set (for a total of 218 test trials), we still did not find any significant increase in the proportion of target looks between pre- and post-naming.
